# Ultrasensitive and Multifunction Plasmonic Temperature Sensor with Ethanol-Sealed Asymmetric Ellipse Resonators

**DOI:** 10.3390/molecules23102700

**Published:** 2018-10-19

**Authors:** Jun Zhu, Jian Lou

**Affiliations:** College of Electronic Engineering, Guangxi Normal University, Guilin 541004, China; zhujun1985810@sohu.com

**Keywords:** plasmonic temperature sensor, high sensitivity, wave filter

## Abstract

In order to improve the low temperature sensitivity of conventional sensors, a plasmonic multifunction temperature sensor with high sensitivity is proposed and investigated systematically in this paper. The sensor consists of two metal layers and two ethanol-sealed elliptical resonators connected to a straight waveguide by two rectangular tubes. We numerically analyzed the transmission characteristics of the Nano-device to assess its performance with the finite element method and achieved great optical properties. The results show that an obvious blue shift of the transmission spectrum appears by varying temperatures, exhibiting a great sensing effect. Sensitivity of the sensor reaches −3.64 nm/°C, far greater than conventional temperature sensors. Our research also demonstrates that the transmission spectrum could be modulated efficiently by the ratio of semi-short axis to semi-major axis of the ellipse resonators and the width of two same rectangular tubes. Furthermore, the Nano-device has a filtering characteristic. The transmittances of pass-band and stop-band are 96.1% and 0.1%, respectively. The results of this study can pave the way for low-cost sensing application in high-density photonic circuits and biosensors.

## 1. Introduction

Surface plasmon polaritons (SPPs) are electromagnetic excitations that tightly bind to the surface of metal, which can limit the energy to the interface of metal and insulator [1,2,3,4]. One of the most fascinating features of SPPs for optical applications is that they are helpful to concentrate and channel incident waves with nanostructures [5]. This attractive characteristic allows researchers to tailor SPPs for practical applications. Depending on the properties of the SPPs, they can be divided into two main fields: (1) A sensing application implemented by the relation between surface plasmon resonance (SPR) and the refractive index, and (2) the enhancement of field intensity and inducement to light with a nanostructure [6,7]. Temperature sensors are one of promising directions in sensing applications, therefore a variety of sensors to detect temperature have been designed. Şahin Kaya Özdemir and Gönül Turhan-Sayan designed a three-interface structure to realize temperature sensing with detection of angular changes caused by temperature [8]. Wang et al. fabricated a nanowire-based temperature sensor with multimode interference and achieved a sensitivity of 11.4 pm/°C [9]. Qiu et al. proposed a temperature sensor using an isopropanol-filling crystal fiber, with a sensitivity reaching −166 pm/°C [10]. Wu et al. built a peanut-shaped structure whose sensitivity reached as high as 0.1 nm/°C at the temperature of 900 °C [11]. These fiber-based nanostructures are more suitable for high temperature environments. However, due to their slightly low sensitivity, they are not a good solution for low temperature applications. In recent years, with the improvement of fabrication process, more research efforts have been put into metallic structures. Yan Kong et al. proposed a sensor, constructed by utilizing a transverse rectangular cavity sandwiched by two metal-insulator-metal (MIM) waveguides. This proposed sensor can monitor temperature with a sensitivity of 0.12 nm/K [12]. A further study proposed a temperature sensor with side-coupled symmetric hexagon resonators, which is based on MIM waveguide and obtained a sensitivity of 0.45 nm/°C [13]. Md. Jubayer Al-mahmod et al. designed a MIM ring structure filled with ethanol, which demonstrated an ability to detect temperature with a sensitivity of −0.52 nm/°C [14]. A typical MIM waveguide with a single defect was utilized for sensing application [15]. This study combined temperature sensing with refractive index sensing, but their study still highlighted room for improvement in sensitivity. The Nano-devices listed above all have different degrees of deficiency in sensitivity at low temperatures (compared to the melting point of most metals), fabrication process, and device volume.

In order to design a great optical sensor, the transmittance, sensitivity at low temperatures, and fabrication processes should be taken into consideration comprehensively. Considering the weaknesses of the above structure, in this paper we proposed a multifunction ultrasensitive plasmonic temperature sensor with two ethanol-sealed asymmetric ellipse resonators. The sensor consists of two ethanol-sealed ellipse resonators connected to a straight waveguide by two rectangular tubes, all of which were filled with ethanol, and two silver layers. The sensitivity of the proposed device increases over an order of magnitude compared with conventional sensors. Furthermore, it also acts as a band-stop filter. The sensor has great potential for multi-purpose application in Nano-devices and situations where high real-time temperature sensitivity is required, such as the environmental monitoring of biochemical reactions.

## 2. Model Structure and Theoretical Analysis

### 2.1. Geometric Structure of the Temperature Sensor

**Figure 1:**
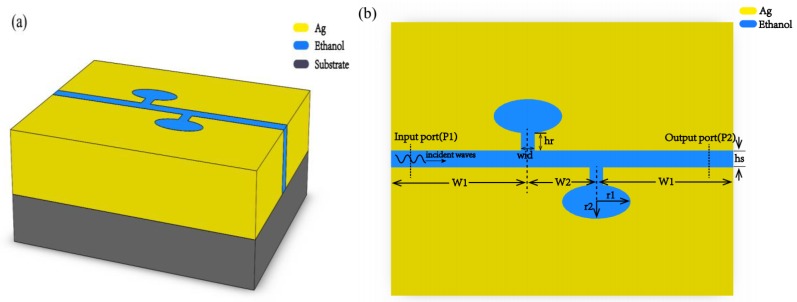
Schematic diagram of the temperature sensor based on metal-insulator-metal waveguides is shown. (**a**) Schematic diagram of the three-dimensional structure. The two layered structure is clearly displayed in the picture, composed of a waveguide layer and a quartz substrate layer. (**b**) The top view of the MIM waveguide layer. The ethanol-sealed cavities (blue in the picture), composed of two ellipse resonators, two rectangular tubes and a straight waveguide, were sandwiched by two silver layers.

As shown in Figure 1, the schematic view of the geometric structure is depicted. The structure is composed of a waveguide layer and a substrate layer. Figure 1a is three-dimension schematic view and 1b is a two-dimension schematic view of the proposed structure. From the three-dimension geometry diagram we can see that a MIM waveguide layers were deposited on the quartz substrate which provided mechanical buffering. Figure 1b, the top view of Figure 1a, clearly shows the structure of the waveguide. Two ellipses resonators, connected to a straight waveguide by two rectangular tubes, were sandwiched between two silver layers. Our reasoning for choosing silver as metallic material was that the imaginary part of relative permittivity is smaller than most metals so it has lower energy-loss compared to other metals [16]. The sensor was easily fabricated. Firstly, a silver layer was deposited on the quartz substrate by a chemical vapor deposition method. Then, the interconnected cavity structure, composed of two elliptical resonators, a straight waveguide, and two rectangular tubes, was etched on the silver film by an electron beam etching method. Finally, ethanol was filled into the interconnected cavity structure by capillary attraction.

We fix the height of the straight waveguide at 50 nm, indicated by the symbol hs in the figure. The height of two identical rectangular tubes was set as 50 nm and the initial width was 40 nm, which is denoted as hr and wid in Figure 1b, respectively. The ratio of semi-short axis (r2) to semi-major axis (r1) of the ellipse resonator is represented as a. The semi-major axis was set as 100 nm. The distance from the middle axis of the left (right) rectangular tubes to the left (right) edge of the metal is W1 and the distance between the middle axes of two rectangular tubes is W2, whose initial value was set as 200 nm.

### 2.2. Theoretical Analysis

The strong ability to confine energy of surface plasmon polaritons beyond the diffraction limit renders MIM structure more attractive for the photonic circuits. In this study, we choose transverse magnetic (TM) waves as incident waves to ensure the excitation of SPPs [17]. The dispersion relation for TM mode in a MIM structure has been previously defined by [18,19,20]

(1) $$\varepsilon_{in}k_{z2}+\varepsilon_{m}k_{z1}\coth\left(-\frac{ik_{z1}}{2}\omega \right)=0$$

where $k_{z1}$ and $k_{z2}$ are given by following equations which come from momentum conservations, respectively.

(2) $$k_{z1}^{2}=\varepsilon_{in}k_{0}^{2}-\beta^{2}$$

(3) $$k_{z2}^{2}=\varepsilon_{m}k_{0}^{2}-\beta^{2}$$

where β is the propagation constant of SPPs, $\varepsilon_{in}=n^{2}$ is the dielectric constant of the insulator, $\varepsilon_{m}$ is the dielectric constant of the metal and $k_{0}=2\pi /\lambda$ is the vector in free space.

As for metal layer, we choose Ag as our meFigtal material because of its low power-absorption. The complex relative permittivity of silver can be characterized by the Drude formula [21]

(4) $$\varepsilon_{m}\left(\omega \right)=\varepsilon_{\infty }-\frac{\omega_{p}^{2}}{\omega^{2}+i\omega \gamma }$$

where $\varepsilon_{\infty }$ = 3.7 is the dielectric constant at infinite angular frequency, $\omega_{p}$ is the bulk plasma frequency with a value of 9.1 eV, ω is the angular frequency of the incident waves and γ=0.018 eV represents the damping frequency (Parameters achieved by processing experimental data [22]). Moreover, the influence of temperature on the dielectric constant of silver can be ignored only at low temperatures excitations [23], indicating that silver is suitable for the type of sensing application required for this paper.

Temperature sensor was realized by manipulating the electromagnetic properties of the SPP, utilizing the change in the refractive index of the temperature sensing material caused by the ambient temperature. In this work, we chose “thermo-optic liquid” ethanol as our sensing material because of its high temperature coefficient of refractive index. Ethanol is a preferable choice for low temperature sensing with a melting point of −144.3 °C and a boiling point of 78 °C. Generally, the relation between the refractive index of liquid sensing material and ambient temperature can be depicted by the following equation [24]:

(5) $$n=n_{\mathrm{liq}}+\frac{dn}{dT}\left(T-T_{0}\right)$$

where dn/dT is the temperature coefficient of refractive index of the liquid sensing material which indicates the sensitivity of the liquid to temperature change, $n_{\mathrm{liq}}$ represents the refractive index at the reference temperature $T_{0}$ and *T* is the ambient temperature. Therefore, it is easy to obtain the relation between the refractive index of ethanol and temperature and the formula is given by

(6) $$n=1.36048-3.94\times 10^{-4}\left(T-T_{0}\right)$$

where $T_{0}$ is 20 °C, which is the room temperature. Equation (6) represents a liner relation between refractive n and ambient temperature. Comparing to the temperature coefficient of ethanol, the influences of temperature on the substrate and silver are negligible.

Furthermore, the impact of the change in refractive index, which is caused by temperature, on the electromagnetic characteristics of the sensor was mainly reflected in the transmittance. In this paper, we systematically investigated the transmission characteristics in the near infrared spectrum. The transmittance was defined as $Tr=P_{\mathrm{out}}/P_{\mathrm{in}}$, where $P_{\mathrm{in}}$ and $P_{\mathrm{out}}$ are the power of the input port (*P*1) and output port (*P*2), respectively. As mentioned above, sensitivity is the most direct indicator of a sensor and the definition of sensitivity is s=Δλ/ΔT, whose unit is nm/°C.

As noted earlier, the design has a function of wave filtering and this sensor can also act as a band-stop filter. The stop-band width was defined as the width of transmittance spectrum below 0.1. It was an important indicator for evaluating the performance of a band-stop filter. The smaller the stop-band, the better the performance. Moreover, we used the steepness of rising edge (SRE) and falling edge (SFE) in transmittance spectrum to describe the ideal degree of the filter. The definition of SRE is given as $SRE=\Delta Tr/W_{r}$ where ΔTr is the difference of *Tr*1 and *Tr*2 and it is usually taken as 0.4, $w_{r}$ and is the difference between the corresponding abscissa to *Tr*1 and *Tr*2 in the rising edge. The unit of SRE is ${\mu m}^{-1}$. The definition of SFE is the same as that of SRE.

## 3. Simulation Analysis of Transmittance

**Figure 2:**
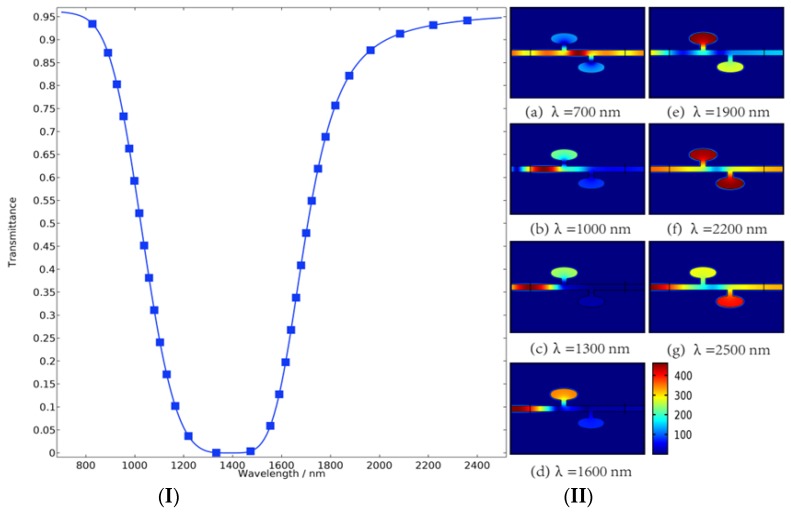
(**I**) Transmittance of the sensor with a = 0.5, wid = 40 nm. (**II**) Under the structural parameters of Figure 2I, magnetic field distribution of the proposed device was shown at the incident waves of different wavelengths ranging from 700 nm to 2500 nm at intervals of 300 nm.

We quantitatively investigated the transmission characteristics of the proposed sensor by the finite element method. As shown in Figure 2, we obtained the magnetic field distributions and the transmittance of the sensor when the width of two rectangular tubes (indicated by the symbol of wid) is set as 40 nm and the ratio of semi-short axis to semi-major axis of the ellipse resonators (indicated by the symbol of a) is set as 0.5. The transmittance can be controlled by varying the wavelength of incident waves, and this significant property provides a solution for tuning the sensor. As we can see from Figure 2I, with the wavelength increasing, the transmittance firstly appears to decrease sharply and maintains low transmittance over a range of wavelengths, followed by a rapid rise. The shape of this transmission spectrum line is very close to an ideal band-stop filter. Figure 2II shows the magnetic field distribution of the sensor at different wavelengths corresponding to the transmission spectrum on the left, from which we can clearly observe the evolution process of magnetic properties of the sensor by varying the wavelength of incident waves. The ellipse resonators couple with straight waveguide efficiently through the rectangular tubes. The ellipse resonator acts like a magnetic container with a containment effect. At the beginning, almost all of the incident waves pass through the waveguide structure and the transmittance exceeds 95%, as shown in Figure 2(IIa). As the wavelength increases, the magnetic field strength of the left ellipse resonator was constantly enhanced. The transmittance is declining in this process. When the magnetic field strength of the left ellipse resonator increases to a certain extent, the magnetic intensity in the resonator on the right starts to increase while the transmittance is rising at the same time. Finally, the transmittance gradually returned to its original level.

**Figure 3:**
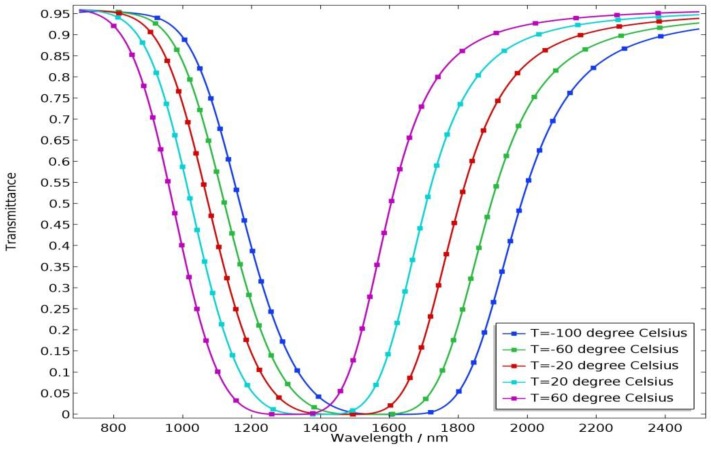
Transmittance of the proposed sensor for different temperatures with a = 0.5, wid = 40 nm.

The mechanism of the temperature sensor is to make use of the influence of ambient temperature on its electromagnetic characteristics and further affect its optical characteristics. Therefore, it is critical to investigate the effects of temperature on the properties of the sensor. Figure 3 shows the transmittance of the sensor for different temperatures with a = 0.5, wid = 40 nm. As the temperature increases from −100 °C to 60 °C with a step of 40 °C, the transmission spectrum displays a blue shift phenomenon and the width of the stop-band decreases. This is because, as the temperature increases, the refractive index of ethanol decreases, thereby weakening the strength of the vertical coupling. This indicates that the sensor is suitable for sensing application for low temperature.

## 4. Sensing Performance

### 4.1. Adjustment of the Ratio of Semi-Short Axis to Semi-Major Axis

**Figure 4:**
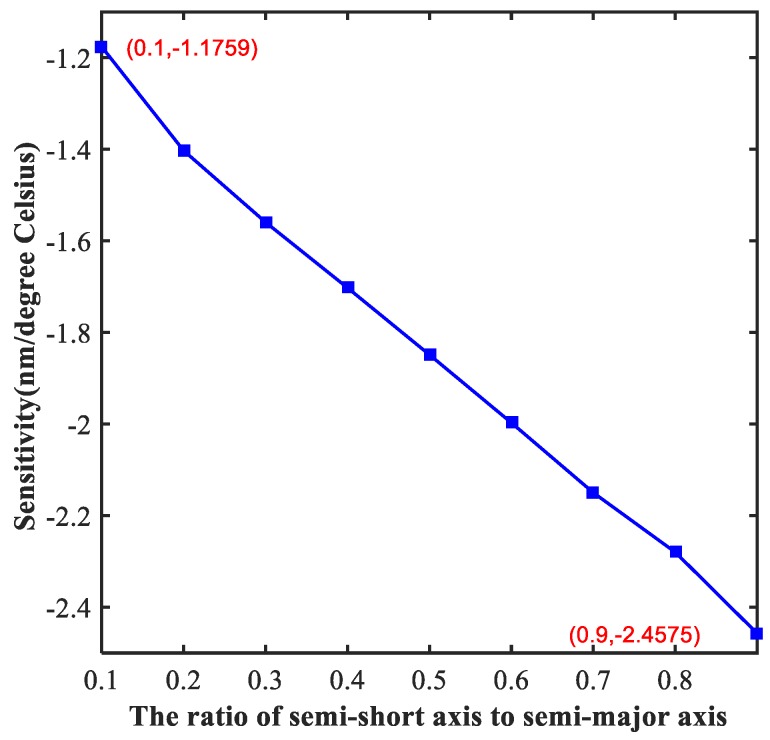
The sensitivity of the proposed sensor with different ratio of semi-short axis to semi-major axis. The maximum sensitivity of the sensor is about −2.45 nm/°C and the minimum sensitivity is about −1.17 nm/°C.

We analyzed the effects of structural parameters on the performance of sensing characteristics of the proposed sensor below. Optimizing the structural parameters is of great significance for the design of Nano-devices, and the highest performance can be obtained through this process. Firstly, we studied the effect of the ratio of semi-short axis to semi-major axis of the ellipse resonators (indicated by the symbol of a) on the sensor. As we can see from Figure 4, as the value of a increases, the absolute value of sensitivity continues to rise. Furthermore, the sensitivity has a linear relation with the value of a, which supplies a great solution for tuning the sensor to meet different requirements for sensitivity. When the value of a is 0.9, the sensitivity reaches the maximum, which is as high as −2.45 nm/°C.

### 4.2. Adjustment of the Width of the Rectangles

**Figure 5:**
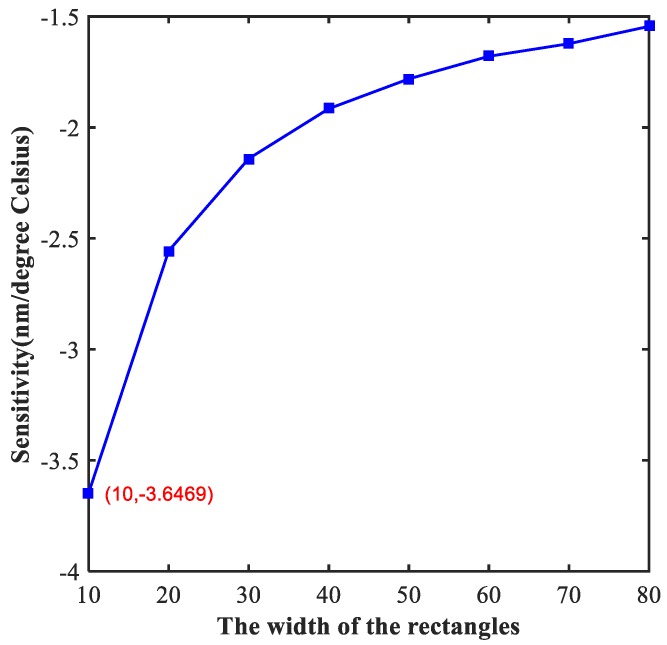
The sensitivity of designed device with different width of the rectangles. The maximum absolute value of sensitivity is taken at 10 nm in the figure, which is marked in red.

In addition, the width of two rectangular tubes (indicated by the symbol of wid) is another important parameter that affected the performance of the proposed device. We focus on the impact of the width of the two small rectangular tubes on the sensor in the following, and the distance from the middle axis of the right rectangular tubes to the right edge of the metal (indicated by the symbol of W2) was set as 300 nm. The relation between sensitivity and wid is shown in Figure 5. Unlike the linear relationship depicted in Figure 4, the functional relationship in Figure 5 is similar to a power function. It is worth mentioning that the sensitivity that we obtained is as high as −3.64 nm/°C when wid = 10 nm.

Finally, we obtained the best performance of sensing when a = 0.9 and wid = 10 nm. As shown in Table 1, we investigated some temperature sensor reported in other studies. It is obvious that the sensitivity of traditional sensors is too low to meet the increasing demand for precision sensing applications. The outstanding sensitivity of temperature sensor in this work reaches up to −3.64 nm/°C, which is greater than previous studies. The sensor in the current study has great potential for application in high-sensitive sensing.

## 5. Filtering Performance

### 5.1. Optimization of the Ratio of Semi-Short Axis to Semi-Major Axis

**Figure 6:**
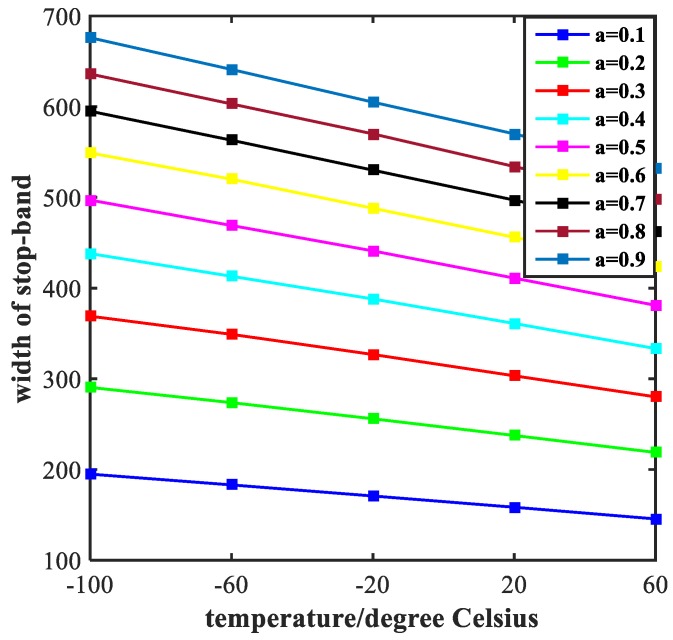
Width of stop-band of the transmittance. The colored lines in the figure represent the width of stop-band of the filter with different values of a.

**Figure 7:**
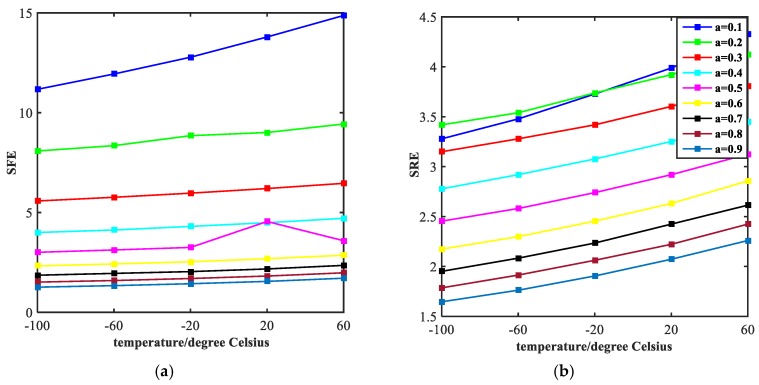
Steepness of the rising edges (SFE) and falling edges (SRE) of the transmission spectrum, corresponding to the different values of a, are plotted in the figure (**a**,**b**) with curves of different colors, respectively.

The width of stop-band and steepness of rising edge (indicated by the symbol of SRE) and falling edge (indicated by the symbol of SFE) are two important aspects of investigating a band- stop filter. Figure 6 exhibits the influence of the value of the ratio of semi-short axis to semi-major axis (indicated by the symbol of a) on the width of stop-band. It is obvious that the width tends to decrease with the increase of temperature. However, from a vertical perspective, the width increases continuously with the increase of the value, ranging from 0.1 to 0.9 at an interval of 0.1. The steepness of rising edge and falling edge of the transmission spectrum are shown in Figure 7. With the increase of temperature, both the SRE and SFE appear to increase with varying degrees. At the same time, as the value of a increases, the SRE and SFE decrease. The trend of each individual line shows that the performance of the filter continues to improve as the temperature increases. We obtained the best performance of filtering at a = 0.1. However, as the value of a increases, the performance of the filter degrades which is opposite to the law of sensing in Figure 4. These results are valuable for our design of multi-purpose Nano-devices.

### 5.2. Optimization of Width of the Rectangles

**Figure 8:**
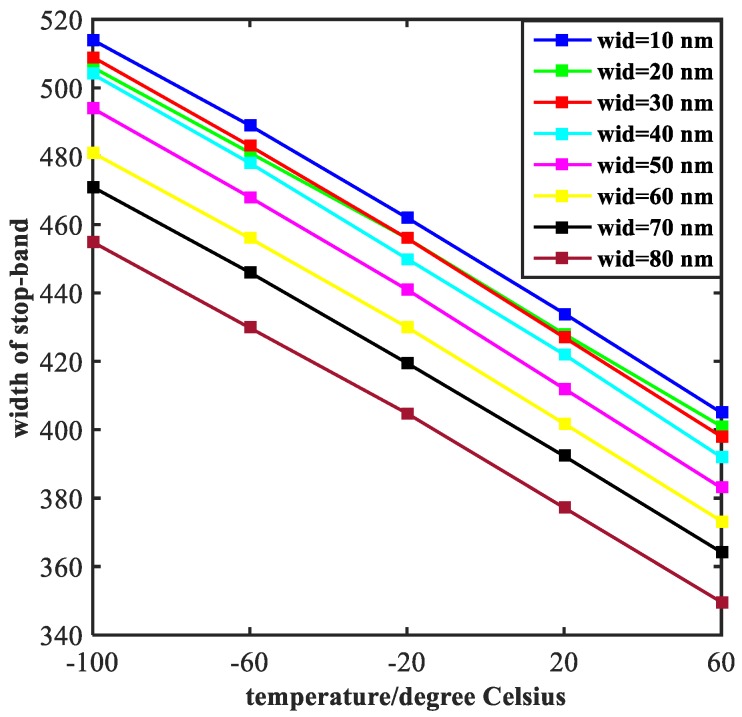
Width of stop-band of the transmission spectrum is shown and the value of wid ranges from 10 nm to 80 nm at intervals of 10 nm.

**Figure 9:**
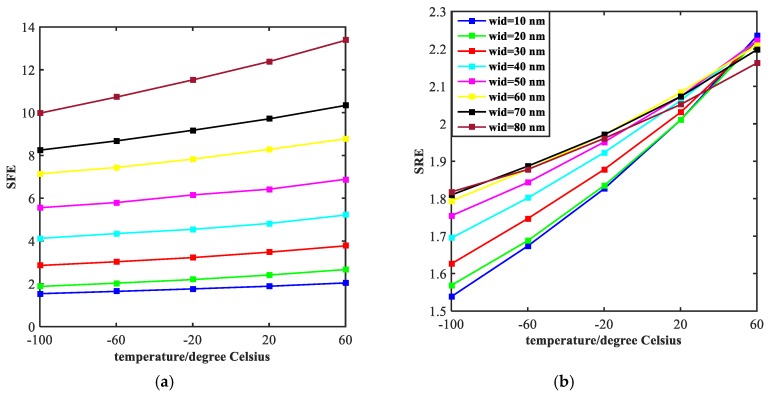
Steepness of the rising edges (SFE) and falling edges (SRE) of the transmission spectrum is shown with curves of different colors in figure (**a**,**b**).

As we can see from the Figure 8, the width of stop-band tends to decline with the temperature increases, which exhibits a similar trend with lines in Figure 6. However, the difference from Figure 6 is that the curves in Figure 8 drop faster. This is because the effect of width of the rectangular tubes (indicated by the symbol of wid) on electromagnetic properties of the structure is much bigger than the ratio of semi-short axis to semi-major axis of the ellipse resonators. The curves of SRE and SFE are plotted in Figure 9. As the temperature increases, both SRE and SFE show an increasing trend. By comparing Figure 9a with Figure 9b, it can be seen that the rising edge is more affected by the width of two rectangular tubes. Moreover, the steepness of the rising edge converges to a point at the end, indicating that the width of the rising edge does not change as the width increases. In general, the performance of filtering is best when wid = 80 nm.

## 6. Conclusions

In this paper, a multifunction ultrasensitive plasmonic temperature sensor with two ethanol-sealed asymmetric ellipse resonators is proposed. The sensor, constructed by utilizing silver as metal layers and an ethanol-sealed insulator layer is sandwiched by the two metal layers. Our study is mainly focused on the transmission spectrum. We systematically analyzed the transmission characteristics to assess the performance of the Nano-device and achieved great optical properties. By properly adjusting the structural parameters of the waveguide, the optimal structural parameters have been obtained. When a = 0.9 and wid = 10 nm, the best performance of sensing was obtained, and when a = 0.1 and wid = 80 nm, the best performance of filtering was achieved. The device is capable of sensing ambient temperature with superior sensitivity, as high as −3.64 nm/°C. It also can be exploited for filtering and exhibits a good performance. The proposed sensor device has a small volume, rendering it promising for the development of miniaturized optical instrument and photonic integrated chip. The sensor also has the advantages of a simple manufacturing process and easy integration.

## Figures and Tables

**Table 1:** The sensitivity of temperature sensors reported in other studies.

Refs	Structure	Temperature Range	Sensitivity
[9]	Optical fiber-tip	0 °C–1200 °C	11.4 pm/°C
[10]	Isopropanol-sealed photonic crystal fiber	23.7 °C–66.1 °C	−166 pm/°C
[11]	Fusion-splicing a peanut-shape structure in fiber	100 °C–900 °C	0.1 nm/°C
[12]	MIM waveguide with a transverse rectangular resonator	250 K–600 K	0.12 nm/K
[13]	MIM waveguide with dual side-coupled hexagonal cavities	−100 °C–60 °C	0.45 nm/°C
[14]	MIM waveguide with ring resonator Structure	−114.3 °C–78 °C	−0.53 nm/°C
[25]	Ethanol-filled photonic crystal fiber	36 °C–55 °C	0.8833 nm/°C
[26]	a selective ethanol-filled photonic crystal fiber	25 °C–33 °C	1.65 nm/°C
This study	MIM waveguide with ethanol-sealed asymmetric ellipse resonators	−100 °C–60 °C	−3.64 nm/°C
